# Challenges of Using Instant Communication Technology in the Emergency Department during the COVID-19 Pandemic: A Focus Group Study

**DOI:** 10.3390/ijerph182312463

**Published:** 2021-11-26

**Authors:** Yuh-Shin Kuo, Chien-Hsin Lu, Po-Wei Chiu, Hung-Chieh Chang, Yu-Yuan Lin, Shao-Peng Huang, Pei-Yu Wang, Cheng-Jen Chen, I-Chen Lin, Jing-Shia Tang, Ying-Hsin Chang, Ray Hsienho Chang, Chih-Hao Lin

**Affiliations:** 1 Department of Emergency Medicine, National Cheng Kung University Hospital, College of Medicine, National Cheng Kung University, Tainan 70403, Taiwan; fanny_kuo5@hotmail.com (Y.-S.K.); jazojazojazo@gmail.com (C.-H.L.); wayne741002@gmail.com (P.-W.C.); robbie1007@gmail.com (H.-C.C.); yuyuan1122@hotmail.com (Y.-Y.L.); brent98001123@gmail.com (S.-P.H.); pat2140@gmail.com (P.-Y.W.); finring.com@gmail.com (C.-J.C.); m1112mm1112m@hotmail.com (I.-C.L.); acls1313@gmail.com (Y.-H.C.); 2 Department of Nursing, Chung Hwa University of Medical Technology, Tainan 71703, Taiwan; tangjeanine@hotmail.com; 3 International Doctoral Program in Nursing, College of Medicine, National Cheng Kung University, Tainan 70403, Taiwan; 4 Department of Security and Emergency Services, Embry-Riddle Aeronautical University-Worldwide, Daytona Beach, FL 32114, USA

**Keywords:** COVID-19, emergency department, focus group study, instant communication technology, patient assessment

## Abstract

A record outbreak of community-spread COVID-19 started on 10 May 2021, in Taiwan. In response to the COVID-19 pandemic, care facilities have adopted various protocols using instant communication technology (ICT) to provide remote yet timely healthcare while ensuring staff safety. The challenges of patient evaluation in the emergency department (ED) using ICT are seldom discussed in the literature. The objective of this study was to investigate the factors influencing the utility of ICT for patient assessment in emergency settings during the pandemic. The patient flow protocol and the ED layout were modified and regionalized into different areas according to the patient’s risk of COVID-19 infection. Nine iPads were stationed in different zones to aid in virtual patient assessment and communication between medical personnel. A focus group study was performed to assess and analyze the utility of the ICT module in the ED. Eight emergency physicians participated in the study. Of them, four (50%) had been directly involved in the development of the ICT module in the study hospital. Three main themes that influenced the application of the ICT module were identified: setting, hardware, and software. The setting theme included six factors: patient evaluation, subspecialty consultation, patient privacy and comfortableness, sanitation, cost, and patient acceptability. The hardware theme included six factors: internet connection, power, quality of image and voice, public or personal mode, portable or fixed mode, and maintenance. The software theme included six factors: platform choices, security, ICT accounts, interview modes, video/voice recording, and time limitation. Future studies should focus on quantifying module feasibility, user satisfaction, and protocol adjustment for different settings.

## 1. Introduction

Instant communication technology (ICT) has been applied to improve healthcare in various innovative modalities [1]. The model of patient care, especially in the emergency department (ED), is changing dramatically in response to the ongoing COVID-19 pandemic [2,3,4]. Achieving adequate patient evaluation while minimizing direct contact with patients to reduce the risk of infection is an important concern.

Several models of delivering healthcare through computer devices have been designed for different settings [5,6,7,8]. The Infectious Diseases Society of America has previously used telemedicine to provide subspecialty care, manage patients with chronic disease, and prevent and control infection transmission [9]. The use of telehealth communication in intensive care unit settings, specifically between physicians and families, is an effective means of communication to avoid direct in-person contact [10]. Telemedicine has been effectively used to reduce physician exposure risks and conserve personal protection equipment (PPE) [3,11]. During the COVID-19 pandemic, telemedicine has been applied in different subspecialties, including neurology [12], orthopedics [13], neurosurgery [14], dermatology [15], and even in ophthalmology [4], for remote consultations and assessment. Family meetings and conversations between patients and families can be carried out by using telecommunication [16].

Different devices can be used for instant telecommunication, including two-way radio transceivers, phones, smartphones, computers, and tablets. Two-way radio transceivers have the advantage of being relatively inexpensive, simple to operate while wearing PPE, and easy to decontaminate after use [17]. However, two-way radio transceivers do not have a secure channel, and there is often miscommunication between personnel sharing the same channel. Radio frequencies are often blocked by lead-lined walls [17]. Both audio and video communications are available on telephone devices, and video consultations are preferred by physicians managing acute conditions [18,19]. When consulting by video, however, technical problems are frequently noted, and the information gained from communication is often inferior to that gained from face-to-face interaction. Therefore, video consultation is more suitable for less complicated clinical problems that do not require physical examination [20].

Of the devices available for video communications, handheld video devices are useful for emergency imaging teleconsultation [21]. Evaluation, diagnosis, and clinical advice can be provided in a distant yet timely fashion by using smartphones [22]. Mobile devices with larger screens are preferred by physicians [23]. In comparison with desktop computers, tablets can minimize the time a physician spends at a computer workstation and can aid in computing at the bedside [24]. The use of tablets for video consultation can improve clinical performance, analysis of clinical information, and communication between physicians and patients [25,26].

Taiwan has seen a record outbreak of community-spread COVID-19 in 2021. The EDs throughout Taiwan were confronted with a surge of patients with risk of infection in May 2021. Prior to this community outbreak, suspected COVID-19 cases managed in the ED were very few in numbers. The ED managed patients with a high risk of COVID-19 infection in negative-pressure isolation rooms, donning and doffing PPE during each encounter. This patient care model was immediately challenged by the increase in suspected cases of COVID-19 infections during the beginning of the outbreak. In response to the pandemic, the model of patient care has changed dramatically, and applications of ICT have been used in several medical environments. During this pandemic, ED workers have faced several challenges. Strategies to reduce exposure risks and to decrease disease transmissions must be made. Patients must be diverted into different areas according to their clinical status and risk of infection to reduce risks of disease transmission. Evaluating and reallocating patients while ensuring staff safety prompted the use of telemedicine in the ED environment.

To overcome the obstacles in patient evaluation in the ED during the COVID-19 pandemic, the ED has utilized an ICT module based on tablets. Application of ICT modules in the ED may be costly and time consuming. There are several aspects to be handled and the infrastructure of the module involves several departments, including information technology teams and medical teams of physicians and nurses. Hospitals and healthcare facilities play a critical role in response to epidemics and must react immediately to the disaster. To act promptly to a communicable disease epidemic, an ICT module can be added to a strategic preparedness and response plan. However, the challenges of patient evaluation in the ED using ICT are seldom discussed in the literature [3,6,27,28]. Introducing a novel module in the ED, especially during a pandemic, can be an arduous experience. For those interested in utilizing ICT modules, avoidance of “reinventing the wheel” is recommended. Identification of the challenges may facilitate the process of ICT setup, reduce the cost of implementation, and improve the satisfaction of stakeholders. Therefore, we conducted this study to explore the factors influencing the development and use of ICT to assess patients in the ED.

## 2. Materials and Methods

### 2.1. Study Setting

This study was conducted in the ED of a tertiary teaching hospital in Tainan City. Tainan has a population of 1.87 million in an area of 2192 square kilometers in southern Taiwan. The average number of daily ED visits was approximately 200 patients, of which approximately 20% were triaged as critical. The ED was staffed with 54 emergency physicians and 157 registered nurses.

An outbreak of community-spread COVID-19 started on 10 May 2021, in Taiwan. One-third of patients presented to the ED with symptoms such as fever, rhinorrhea, cough, ageusia, or anosmia, which poses a certain risk of COVID-19 infection. The rapid screening test to detect COVID-19 infection was not widely available at the time. We adjusted our workflow and operation system in response to the surge in suspected COVID-19 cases. Triage, redistribution, reallocation, and evaluation of patients while ensuring the safety of the staff were of utmost importance. Strategies to minimize exposure risks included redesigning the ED into different zones, patient diversion, and the use of ICT.

### 2.2. Regionalization within the ED

**Figure 1:**
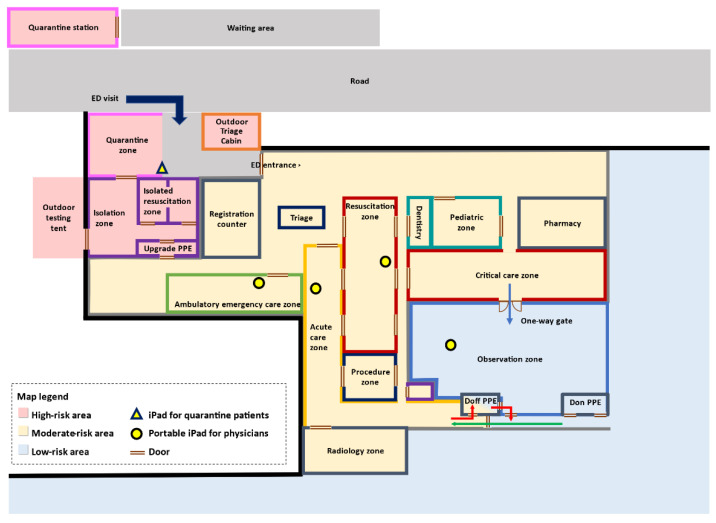
Modified floor plan during the COVID-19 pandemic. The ED was sectioned into three main areas: high-risk (pink), moderate-risk (yellow), and low-risk (blue) areas. High-risk areas included an outdoor triage, a quarantine zone, a quarantine station, and an isolation area that was composed of an isolation zone, two isolated resuscitation rooms, and a room to don and doff PPE. The moderate-risk area included an indoor triage, a resuscitation zone and a critical-care zone for critically ill patients, an ambulatory emergency care zone, and an acute care zone. The low-risk area (blue) consisted of an observation zone, where patients with negative COVID-19 PCR tests awaited admission or further management, and rooms to don and doff PPE. The blue arrow shows a one-way path to reallocate patients from moderate-risk areas to low-risk areas. The green arrow shows the direction of the staff flow to don PPE. The red arrow shows the direction to doff PPE and leave the ED. The iPad in the quarantine zone was placed on an immobile tablet security stand (triangle), and two iPads were given to physicians in the resuscitation zone, ambulatory emergency care (AEC) zone, acute care zone, and observation zone (circles).

To segregate non-COVID-19 cases from suspected cases, all patients who visited the ED were diverted to separate areas according to their risks of potential COVID-19. We therefore regionalized our ED as high-risk, moderate-risk, and low-risk areas. Figure 1 shows the modified floor plan of the ED during the pandemic.

In the high-risk area, we demarcated four spaces: an outdoor triage, a quarantine zone, a quarantine station, and an isolation sector that consisted of an isolation zone and an isolation resuscitation zone. Ambulatory patients with high-risk and stable vitals were diverted to the quarantine sectors. Across the quarantine zone, a quarantine station was set up. The quarantine station, designed to relieve the surging ED workload, was a structured building with 18 isolated compartments and was operated by a separate team. The isolation sector consisted of an isolation zone and two negative-pressure isolated resuscitation rooms. The isolation zone was designed to allocate high-risk, non-ambulatory patients with stable vitals. Those at high risk and in need of emergency critical care were placed in negative-pressure resuscitation rooms.

The moderate-risk area included an ambulatory emergency care zone, an acute care zone, a resuscitation zone, and a critical-care zone. The acute care zone, with a capacity of 12 beds, was used to manage non-ambulatory patients. The resuscitation zone and critical-care zone had space for 36 beds.

All patients were tested with rapid COVID-19 polymerase chain reaction (PCR) tests. The low-risk area, composed of an observation zone and dressing rooms to don and doff PPE, was used to allocate patients with negative COVID-19 PCR tests who were awaiting admission or further management. This zone had a capacity of 30 beds.

Different levels of PPE were required for staff according to the risk level. The PPE had to be changed when the medical staff moved from one zone to another.

### 2.3. Patient Flow and the Diversion Protocol

**Figure 2:**
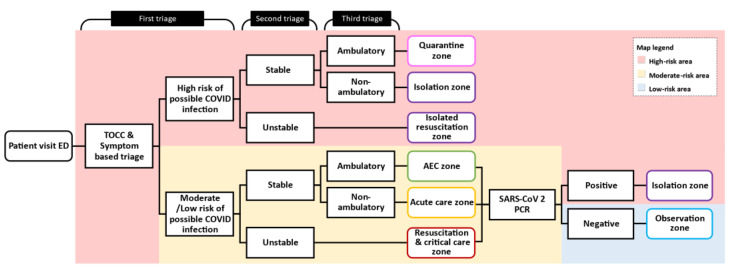
Patient flow and protocol. We modified a triple triage protocol to stratify patients’ risks of COVID-19 infection and diverted them into different areas accordingly. Abbreviations: Acute emergency care zone (AEC).

All patients who visited the ED were diverted to the designated areas according to their risk of COVID-19 infection. To accomplish this diversion policy and to streamline the process, the patient flow was modified into a triple triage protocol: (1) the first triage in the outdoor triage area to screen each patient’s history of travel, occupation, clusters of contacts, and symptoms; (2) the second triage to assess the patient’s vitals; and (3) the third triage to evaluate the patient’s ambulatory status. The protocol of patient flow is shown in Figure 2.

The first triage was set up outside the ED entrance. All ED patients first underwent TOCC (travel, occupation, contact, and cluster) and symptom-based triage. Patients who had positive TOCC, fever up to 37.5 °C within the last three days, or any two of five symptoms (cough, rhinorrhea, diarrhea, lethargy, or anosmia) were triaged as high-risk. The purpose of the outdoor triage with TOCC and symptom-based triage was to segregate high-risk patients from others to prevent disease spread and to remind staff to take appropriate precautions. After the first triage, the patient was diverted to either the high-risk or the moderate-risk area and proceeded to step two, the second triage.

The second triage took place in the outdoor triage if the patient was of high-risk and indoors if the patient was of moderate/low-risk. The purpose of the second triage was to evaluate whether the patient was stable. Patients who had either an ambient oxygen saturation below 92% or a shock index (defined as the heart rate (beats/minute) divided by systolic blood pressure (mmHg) greater than 1 were defined as unstable. High-risk patients who were unstable were placed in the isolated resuscitation room for urgent medical management. Moderate/low-risk patients who were unstable were placed in the resuscitation bay or critical-care zone. Those who were stable proceeded to step three, the third triage.

The third triage was to evaluate the stable patients’ ambulatory status. During pandemics, the number of stable patients who still need ED care can be exceedingly high, so the third triage was essential to divert the ED patient flow. In the high-risk area, stable and ambulatory patients were placed in the quarantine zone; non-ambulatory patients were placed in the isolation zone. In the moderate-risk area, stable and ambulatory patients were allocated to the ambulatory emergency care zone; non-ambulatory patients were placed in the acute care zone.

Patients with moderate/low-risk were treated and managed in the ED. All patients were tested with rapid COVID-19 PCR tests. Those who had negative rapid COVID-19 PCR test results were defined as low-risk and subsequently reallocated to the observation zone in the low-risk area.

### 2.4. The ICT Module

Our information technology team deployed nine iPads (iPad 8th generation with iPad OS 14 system and Wi-Fi, Apple Inc., Cupertino, CA, USA) in the ED, one in the quarantine zone and two in each other zone (Figure 1). Each iPad was equipped with FaceTime (Apple Inc., Cupertino, CA, USA), which provided the ability to make free audio or video calls. We composed and provided an instruction manual to our staff. The iPads in the different zones were mobile and handled by physicians in each zone, while the iPad in the quarantine zone was placed on a stand.

**Figure 3:**
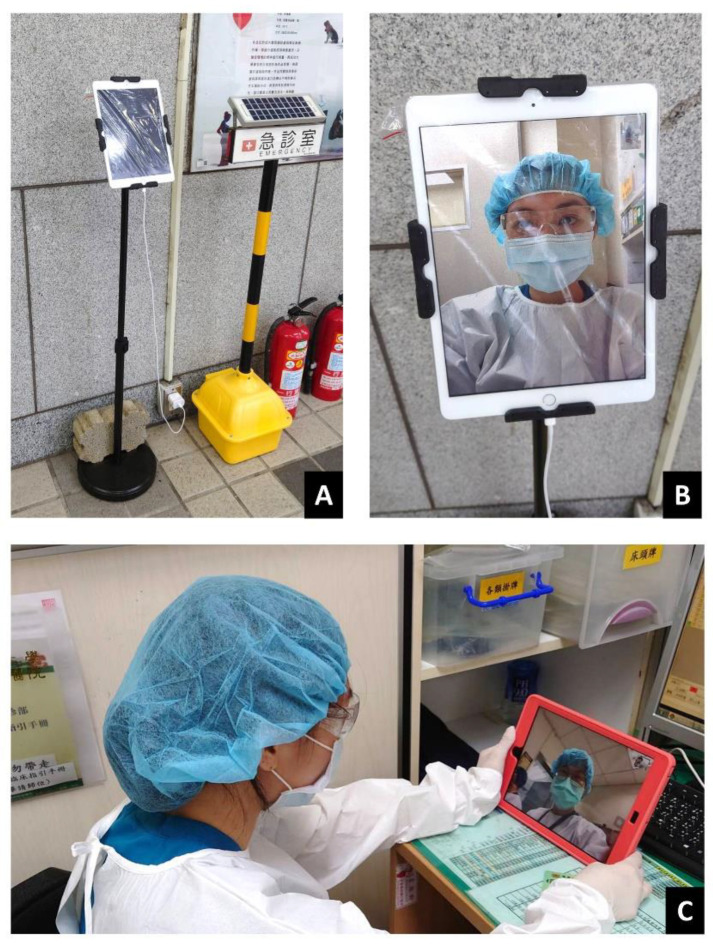
iPad in the quarantine zone. (**A**). The iPad in the quarantine zone was placed on an immobile security stand and covered with a transparent plastic wrap, which was regularly changed, and the tablet was sanitized after each encounter. (**B**). This picture shows the patient’s view of the tablet in the quarantine zone when a physician initiated virtual communication via FaceTime. (**C**). Virtual communication between physicians in different zones could be achieved with iPads and FaceTime. The photo consent was obtained.

The patient-side iPad was in the quarantine zone. The iPad was placed on a tablet security stand (Figure 3), covered with a transparent wrap, and anchored in the quarantined area. The iPad in the quarantine zone was disinfected with 70% alcohol sanitizer after each encounter, and the transparent cover was changed regularly.

The physician-side iPads were in the resuscitation zone, acute care zone, ambulatory emergency care zone, and observation zone. Patient handover between different zones or work shifts could also utilize ICT communication to reduce contact between medical personnel. With the iPad equipped in each area, physicians could initiate either audio or video communication, thereby obtaining the patient’s history and presenting illness without direct contact. Explaining examination results and discussing treatment plans among physicians, patients, and their families could be achieved through this ICT module.

### 2.5. Focus Group Study

As we discussed previously, the purpose of conducting this research was to explore the factors influencing the development of the ICT to assess patients in the ED. Consequently, we employed a qualitative approach to design this study. A qualitative approach is preferable for investigating the quality of a social phenomenon as it provides rich discussions and descriptions considering the research question we proposed [29,30,31].

More specifically, we conducted focus group interviews to gain insights and understanding from those physicians who utilized the ICT (see Appendix A for the interview guide). The value of a focus group is its ability to enable the research team to further understand those key factors influencing the effectiveness of applying the ICT during the COVID-19 pandemic. Moreover, it provides a more comprehensive approach to further explore those common themes and concepts mentioned by our interviewees. In this research, a single-disciplined focus group study was conducted two weeks after the implementation of patient evaluation using ICT. A homogeneous focus group facilitates information sharing and the process of reaching conclusions [32]. Participants were recruited from a population of 54 emergency physicians.

Following the principles of conducting a qualitative study, the research team utilized the standard case sampling method, which reflects the “average-like sample” in the selected hospital [31]. Consequently, the research team determined the number of participants, including the moderator, which was set to be between six and ten. Of those participants, it was determined that half would come from being directly involved in the development of ICT modules in the study hospital and the other half would come from having had experience using the ICT module. Participation was voluntary, and no financial reimbursement was provided. Participants were free to drop out at any time.

The focus-group discussion had three sessions and was conducted in June 2021. Each session lasted approximately 1–1.5 h. The sessions were conducted every other day and were led by an experienced moderator. The discussions were internet-based, using the video-conference application Google Meet (Google Inc., Mountain View, CA, USA). To facilitate the data collection and analysis, a member of the research team took notes during every discussion. These notes were then shared with all participants after those interviews to ensure they reflected the discussions and insights of the group interviews.

As a result, the research team analyzed the notes and grouped those common themes and concepts manually together. Since there were a relatively small number of interview notes, and there was little previous research relevant to this subject, the researchers selected the grounded theory approach to analyze the data [33]. The researchers utilized the open coding process to identify concepts and themes within the interview notes and later connected them to develop a complete analysis. More specifically, researchers first performed an inductive thematic analysis of the data, including coding and categorizing the data. During this process, data were thematically analyzed using the theoretical framework of acceptability of healthcare interventions to assess the acceptability they experienced from the perspective of implementation delivery [34].

To enhance the validity of this research, the research team met every week to discuss the definitions of each code and compare the results from the data collection. By cross-coding the same qualitative data, the research team was able to merge certain codes and develop new codes. This method, peer debriefing, ensures the coding processes would reflect the insights and consensus from all researchers, and not merely from individual perspectives [35]. Those themes and factors generated from the data analysis were then reviewed by the research team, and similar ideas and concepts were merged to reach a final consensus.

## 3. Results

Eight emergency physicians, with the highest education level of Doctor of Medicine, participated in the study. None of the participants dropped out. Of them, four (50%) had been directly involved in the development of the ICT module in the study hospital. Two (25%) of the participants were female. The age and working experience of the participants ranged from 28 to 48 years and 3 to 20 years, respectively.

Three main themes were identified in their considerations on using ICT for patient assessment in the ED: setting, hardware, and software.

The first theme, “setting”, has six major factors: patient evaluation, consultation with subspecialty, patient privacy and comfortableness, sanitation, cost, and patient acceptability. Important considerations regarding the setting are summarized in Table 1.

The second theme, “hardware”, has six major factors: Wi-Fi/internet connection, power, image and voice quality, public/personal modes, portable/fixed modes, and maintenance (Table 2).

The third theme, “software”, has six major factors: platform choices, security, ICT accounts, interview modes, video/voice recording, and time limitation (Table 3).

## 4. Discussion

Our study identified several important factors in deploying an ICT module to assess patients in an ED. We found that ICT may be an option for evaluating patients presenting with mild symptoms and could be an important way to reduce infection risk during the pandemic.

The pandemic has significantly transformed the concept of telemedicine. Before the COVID-19 pandemic, telemedicine was commonly applied to chronic and stable patients in remote areas or less accessible settings. For instance, telemedicine was applied in home palliative hospice care to reduce emergency department visits [36]. Telemedicine was efficiently used in monitoring chronic conditions such as management of heart failure or blood sugar control consultations in diabetic patients [37]. Telemedicine was also widely utilized in prehospital settings, such as patient interviews and consultations, patient triage, and telemonitoring. In prehospital settings, synchronous video communication aided in providing the call dispatcher with more information on the scene when handling emergency calls [38]. In contrast to traditional remote telemedicine in outpatient settings, the purpose of applying ICT in the ED during the COVID-19 pandemic was to reduce direct contact between healthcare providers and patients and thus minimize disease transmission risk.

During the pandemic, we modified and regionalized our ED into different areas and zones according to the patient’s risk of COVID-19 infection. Different levels of PPE were suggested for different areas and had to be changed if personnel were to move from one area to another. Communication between medical staff in different areas who were wearing full PPE was a challenge. However, this was largely resolved by the implementation of the ICT module.

We found the use of ICT for patient assessment was more suitable for ambulatory patients that presented with mild symptoms. Although ICT was useful for history taking, general appearance assessment, and collecting information from patients’ families that were not present in the ED, most emergency physicians were less comfortable performing physical examinations using ICT due to previous practice habits.

The internet signal and bandwidth connectivity influenced the quality of images and video, which thereby impacted the effectiveness of ICT. Our hospital provided a stable, low-cost, and high-quality internet connection. However, different hospital settings may pose challenges in this area. A stable, wide-bandwidth, high-quality internet or Wi-Fi connection was essential to make the ICT module workable. Hospitals located in rural areas or those that have lower levels of specialty care may face challenges in providing stable and sufficient internet connection and maintenance. Choice of communication applications and platform was based on the tablet’s operating system and user familiarity. The device and platform used in our ICT module were iPad and FaceTime since their dominant market share might diminish utility barriers, especially among patients. Sanitation was achieved by regularly changing the covering wraps on the tablet and disinfecting the device with 70% alcohol sanitizer after each encounter [39].

We faced several challenges when setting up the ICT module in our ED. Some challenges we found in the setting themes were application of ICT in the evaluation of disabled patients or those with language barriers, protecting patient privacy, and the familiarity of ICT use in elder patients. Elder patients and those with a lower socioeconomic status may be less familiar with the use of tablets and can negatively affect the efficiency of virtual patient evaluation. Those who were elderly, disabled, and lacked digital literacy were less able to access computer devices.

Telemedicine platforms seldom aid in communication for patients with hearing, visual, or cognitive disabilities. ICT accessibilities for disabled patients should be taken into consideration and enforced [40]. To overcome this obstacle, when designing protocols in the future, hearing impairments and other disabilities should be taken into consideration. Communications between physicians and patients with hearing disabilities or language barriers could be performed via ICT that has add-on functions of lip reading, sign language, or real-time audio interpretation.

Another challenge we faced concerning software aspects was patients’ cybersecurity. Security checks and upgrades were performed regularly by our information technology team to prevent personal information breaches. However, not all hospitals or EDs have an information technology team. Maintenance service and efforts must be taken into consideration when planning an ICT module.

Our module required both the patient and physicians to learn how to use the iPad and FaceTime, so we provided an instruction manual. An ICT module should be set up on a regular basis. Medical staff should use this module more frequently to evaluate patients and practice communicating with each other to such an extent that all staff members are familiar with the module. Hence, when there is an outbreak of an infectious disease, the module can be useful to all staff members and shorten the accustoming time.

Our study had several limitations. First, this study was conducted during the initial phase of community outbreak and the resource of rapid diagnostic tools for COVID-19 was extremely scanty. The ED workflow and regionalization could have been extensively modified with a different model should the rapid screening tests have been readily available. Second, we did not evaluate the patients’ perspectives. The comfortableness of medical and nursing staff using tablets for communication was not addressed in our study as well. Questionnaires on satisfaction of using ICT will be provided in the future. Third, prehospital settings were not included in our investigation. However, with appropriate devices and software applications, our protocol can be applied to connect physicians and nonphysicians, such as medical staff in nursing homes or emergency medical technicians. Finally, we used grounded theory in this study because as an exploratory method, grounded theory is suitable for investigating issues that have attracted limited prior research attention. As a method that has advanced over time, grounded theory is a broadly applied research method that has incorporated several different variants [41]. Our study was a pilot study focusing on the challenges of using ICT in the EDs during pandemics. Although digital expertise and ICT have rapidly advanced, there is a disparity in the attitudes and competence levels towards technology among different populations. During the pandemic, the pace of ED workflow changes and adjustments have been expeditious, adding to the difficulties of this study. Although our study interviewed a limited number of practitioners, we believe the results of our study will facilitate the application of communication technology in medical scenarios and could be valuable for future researchers.

## 5. Conclusions

We identified several important considerations that should be addressed in the future development of ICT modules for ED patient assessment, categorized under the setting, hardware, and software. Studies that quantify the utility and feasibility of this module, along with patient and staff satisfaction, should be investigated. With adjustments to the protocol, the ICT module could be applied in prehospital or critical-care settings to provide more thorough, contact-free healthcare.

## Figures and Tables

**Table 1:** Important considerations regarding the setting when using instant communication technology for patient assessment in an emergency department.

Theme: Setting	
Factors	Considerations
Patient evaluation	Patient identity should be verified through electronic, paper-based, or virtual registry systems.
	Patients with mild symptoms are suitable for the use of instant communication technology (ICT) in the emergency department (ED). The door-to-patient-evaluation time and the length of ED stay could be reduced.
	ICT is useful in history taking and general appearance assessment. Emergency physicians were less comfortable performing physical examinations using ICT. Nevertheless, certain physical examinations could be omitted to avoid direct contact.
	Clinical information could be collected from patient families and caregivers who were restricted from entering the ED due to hospital policy.
	The use of ICT could be challenging for disabled patients or those who had language barriers.
Consultation with subspecialty	ICT is acceptable for certain consultations with subspecialties, especially when the clinical judgment is largely based on verbal discussion, such as history taking or shared decision-making.
	The interpretation of imaging studies is generally acceptable.
	The visual evaluation of skin lesions is acceptable sometimes.
Patient privacy and comfortableness	Shelters or booths may be provided to protect patient privacy.
	The surrounding light should be adjusted to achieve better visualization of the screen image.
	The environment should be well ventilated, with the temperature and moisture monitored.
Sanitation	Non-contact- or motion-activated communication applications should be considered to lessen the risk of contact infection.
	A disposable material for activation of applications on tablets with touch screens could be used.
	Most tablet computers can be sanitized using 70% isopropyl alcohol wipes. Aerosol sprays, bleaches, abrasives, or direct-spray cleaners are usually unacceptable. Moisture should be kept out of any opening. One of the simplest ways is to place the tablet into a sealed plastic bag and regularly sanitize the outer surface of the bag with medical alcohol.
Cost	The potential costs included those for technology teams, hardware, software, Wi-Fi or internet connection, etc. To offset these, the cost of PPE could be significantly reduced.
	The regulations on health insurance reimbursement should be clarified.
	Registration for and utilization of instant communication applications may have membership fees.
Patient acceptability	Patient characteristics may be associated with patient acceptability of telemedicine evaluation. We observed that patients who were younger or were using smartphones were more comfortable receiving ICT evaluation.
	We were unsure whether using instant communication applications, compared with traditional face-to-face interviews, would affect the physician-patient rapport.
	The familiarity of the ICT evaluation system among working personnel may have an impact on patient acceptability.

**Table 2:** Important considerations regarding the hardware when using instant communication technology for patient assessment in an emergency department.

Theme: Hardware	
Factors	Considerations
Wi-Fi/internet connection	A stable, low-cost, wide-bandwidth, high-quality internet connection is essential.
	Using Wi-Fi, rather than a fixed internet connection mode, is more practical for the mobile setting of instant communication technology (ICT) evaluation.
Power	The tablets should have a reasonable sustainable source of power for the high usage of instant communication applications. Power that will last for at least one workload shift (usually 12 h) is ideal.
	Extra power-recharging devices should be available.
Image and voice quality	Adequate color presentation, image resolution and size, and voice quality were paramount for patient assessment when using ICT.
	The device should have high-resolution cameras to provide video and image quality. Tablets with built-in front and back cameras were preferred, to enable switching between users.
	The loudness of the voice should be adjustable to provide adequate audio ability and patient privacy.
Public/personal modes	Some health providers may be unwilling to use personal devices as tools for the ICT assessment of emergency patients. A disrupted boundary of professional and personal lives was the major concern.
Portable/fixed modes	When the zoning or regionalization of the emergency department is well set up, a fixed model of patient assessment using ICT is generally feasible.
	Portable devices may be suitable for discussions for shared decision-making, especially when the emergency department is overcrowded. However, portable devices may require more maintenance since they are vulnerable to frequent usage.
Maintenance	The cost and resources needed for maintenance should be preplanned.

**Table 3:** Important considerations regarding the software when using instant communication technology for patient assessment in an emergency department.

Theme: Software	
Factors	Considerations
Platform choices	Instant communication applications work on certain platforms; for example, FaceTime is generally for iOS, while LINE is cross-platform. The choice of applications and platforms should be based on the overall design of the telemedicine system that will use instant communication technology (ICT).
Security	Cybersecurity is important for patient evaluation, so the utilization of ICTs and internet connections should be carefully examined. The risks of personal information breaches were a concern.
	The security upgrades and updates of ICT should be checked regularly.
	End-to-end encryption may provide better protection of conversations between devices. The content of calls may be retrieved and stored on the servers of ICT applications, so the policies of the ICT software should be clarified.
ICT accounts	A single account of emergency department, rather than personal accounts, for each device was preferable.
	Each device may need an individual account for identification.
Interview modes	Instant communication applications that support both one-on-one interviews and chat rooms are preferred. Group chatting is useful for consultations that involve multiple subspecialists, team resource management, or patient family meetings for clinical decision-making.
Video/voice recording	Health insurance reimbursement may require video/voice recording of the patient assessment.
	The recording materials may play an important role when conflicts are encountered, especially medical-legal issues. The accessibility and security control of the storage material should be strictly regulated.
	The storage of video/voice recording requires significant storage space and cost.
Time limitation	Instant communication applications may have time limitations for each call. Extension of the call duration may require additional cost to upgrade the application.
	Time limitations may facilitate the efficiency of patient assessment but should be well communicated between the physician and the patient in advance.

## Data Availability

Not applicable.

## Abbreviations

ED	Emergency department
ICT	Instant communication technology
PCR	polymerase chain reaction
PPE	personal protection equipment
TOCC	travel, occupation, contact, and cluster

## Appendix A. The Interview Guide

1. Do you have experience on using the ICT to communicate with other people?
  - If yes, what are the obstacles on using the ICT?
2. What are the major factors influencing the usage of the ICT?
3. What are the software and hardware you will use with the ICT to facilitate communications?
  - What are the factors that would influence your selection of these software and/or hardware?
4. Do you have any experience on using the ICT to communicate with your patience in hospitals?
  - If yes, what are the major factors contribute to the usage of the ICT in the field of Emergency Medicine (EM).
  - If yes, what are the obstacles on using the ICT in the field of EM.
  - If yes, what are the pros and cons of using the ICT in the field of EM during the COVID-19 pandemic?
    - Anything you think should be improved to better using the ICT in the field of EM during this pandemic?
5. From your perspectives, how does the ICT contribute to the communication between physicians and patients?
